# Health promotion as relational, practical, and structurally constrained work in forensic psychiatric care: a qualitative study on registered nurses’ experiences

**DOI:** 10.1080/17482631.2026.2657694

**Published:** 2026-04-10

**Authors:** Annette Björk, Jelwa Basim, Maria Sundberg, Lars Hammarström, Lisbeth Kristiansen

**Affiliations:** a Department of Nursing, Mid Sweden University, Sundsvall, Sweden; b LARO Psychiatry, Hässlehol and Höör, Sweden; c Forensic Psychiatric Clinic, Region Västernorrland, Sundsvall, Sweden

**Keywords:** Forensic psychiatric nursing, health promotion, nurses’ experiences, qualitative study, recovery-oriented care, relational practice

## Abstract

**Background:**

Forensic psychiatric care involves balancing treatment and rehabilitation with strict legal and security requirements. Registered nurses play a central role in promoting health and supporting patients’ everyday life in this context; however, their perspectives on health-promoting work within coercive institutional settings remain underexplored.

**Aim:**

To explore registered nurses’ experiences of promoting health and supporting everyday life in forensic psychiatric care.

**Methods:**

A qualitative design with individual face-to-face semi-structured interviews was used. Fifteen registered nurses working in forensic psychiatric care in Sweden participated. The interview data were analysed using reflexive thematic analysis following Braun and Clarke, informed by relational perspectives on care and institutional power.

**Results:**

Three interrelated themes were created: (1) *Dialogue as a foundation for participation*, (2) *Everyday practice as a vehicle for recovery*, and (3) *Navigating paradoxes within restrictive care*. Registered nurses described dialogue as foundational for building trust, fostering motivation, and enabling patient participation while simultaneously being shaped by coercive conditions and institutional constraints. Everyday practices, including physical activity, shared meals, and structured routines, functioned as concrete vehicles for recovery, yeat remanined embedded within secitity oriented frameworks. Nurses also described ongoing tensions between health promotion, medication related side effects, and institutional risk management.

**Conclusions:**

This study suggests that registered nurses in forensic psychiatric care experience health promotion as relational, practical, and structurally constrained work within coercive institutional settings. Rather than functioning as a discrete intervention, health promotion appears as an embedded and negotiated aspect of everyday nursing practice. While registered nurses seek to foster participation and well-being, their efforts unfold within institutional arrangements primarily organised around security, risk management, and pharmacological stability. Strengthening organisational support and interprofessional collaboration may enhance the conditions for sustainable health-promoting practice in forensic psychiatric care.

## Introduction

Forensic psychiatric care is characterised by a fundamental tension: it is expected to provide treatment and rehabilitation while simultaneously maintaining security and managing risk. In Sweden, this dual mandate is regulated by the Act on Forensic Psychiatric Care (SFS 1991:^1129^), and the system comprises roughly 25 clinics with around 1,230 inpatient beds across three levels of security. Recent research confirms that this balancing act between care and control is a defining feature of forensic mental health services, in which professionals must continually navigate ethical tensions, institutional constraints, and recovery-oriented aims (Merkt et al., 2021; Tully et al., 2024). Because forensic psychiatry operates at the intersection of medicine and law, it is guided not only by coercive legal frameworks but also by the fundamental rights to health and dignity established in the Health and Medical Care Act.

Furthermore, this dual governance shapes the organisation and delivery of care, in which rehabilitation in forensic psychiatric settings is commonly described as a prolonged and highly structured process aimed at improving patients’ functional capacity and overall well-being while also addressing the risk of reoffending (Lutz et al., 2022; Thomson & Rees, 2023).

Patients in forensic psychiatric care in Sweden are typically men who have been convicted of violent crimes and diagnosed with severe mental illness, such as schizophrenia or other psychotic disorders, often in combination with substance use disorders (Crump et al., 2013; Sivak et al., 2023). Many also live with neurodevelopmental conditions such as ADHD and autism spectrum disorders, as well as intellectual disabilities, which may affect concentration, impulse control, and motivation, thereby limiting opportunities for participation in health-promoting activities (Hofvander et al., 2023; Wyler et al., 2024). These clinical and cognitive complexities shape the conditions under which health promotion can be enacted in forensic psychiatric care.

People living with severe mental illness experience substantial health inequalities, including a 10–20-year reduction in life expectancy largely due to preventable somatic conditions such as cardiovascular disease, diabetes, and respiratory illness (Chan et al., 2023; Walker et al., 2015). Within forensic psychiatric settings, these challenges may be intensified by institutional routines, restricted autonomy, and medication-related side effects (Sariaslan et al., 2022). Contributing factors include smoking, poor diet, and sedentary lifestyle, and adverse effects of psychotropic medication, all of which may be difficult to address within highly structured institutional settings.

The restrictive nature of forensic psychiatric environments may exacerbate these challenges, limiting opportunities for patients to make health-promoting choices and engage in everyday activities conducive to well-being. These conditions underscore the importance, as well as the complexity, of structured health promotion in forensic psychiatric care (Patel et al., 2018; World Health Organisation Regional Office for Europe, 2025).

Nurses play a central role in forensic psychiatric care, as they are the professionals who spend the most time with patients in everyday clinical practice. Their responsibilities extend beyond medical treatment and rehabilitation to include fostering therapeutic relationships, supporting recovery, and promoting health within a restrictive care context (Hellzén et al., 2023; Salzmann-Erikson et al., 2016). At the same time, nurses are required to enforce institutional rules, monitor risk, and uphold security regulations. This dual position situates nurses at the intersection of care and control, where relational engagement and ethical attentiveness coexist with responsibility for surveillance and restriction (Hem et al., 2018).

Previous research has shown that such conditions generate ongoing ethical tensions, requiring nurses to negotiate trust, responsibility, and professional credibility within coercive frameworks (Hammarström et al., 2019; Sollied & Kvande, 2025). Encounters in forensic settings may evoke vulnerability, frustration, and moral uncertainty, highlighting that relational work in this context is neither neutral nor unproblematic. These tensions fundamentally shape how possibilities for recovery and health promotion are understood and enacted in everyday practice. Similarly, research has shown that nurses strive to humanise care within coercive environments, while continuously negotiating safety, risk, and patient autonomy (Sollied & Kvande, 2025; Tomlin et al., 2018). These findings suggest that nursing practice in forensic settings is shaped by ongoing ethical and relational tensions rather than stable or straightforward professional roles. In addition, conceptual work on recovery in forensic psychiatry indicates that patients face distinctive challenges, including long-term institutionalisation, stigma, and the need to integrate their criminal offence into personal narratives (Lumén et al., 2024).

Taken together, these studies indicate that health-promoting nursing practice in forensic settings is inherently shaped by ethical tensions, institutional constraint, and negotiated responsibility. Despite growing attention to health promotion and recovery-oriented care in forensic psychiatry, existing research has predominantly focused on treatment outcomes, risk management, and organisational conditions. Far less attention has been directed towards how nurses themselves understand and enact health promotion within institutional contexts characterised by coercion, security demands, and asymmetrical power relations. As a result, the moral, relational, and structural dimensions of health-promoting nursing practice in forensic settings remain insufficiently explored.

To deepen the interpretation of these tensions, this study draws on selected concepts from Kari Martinsen’s philosophy of care. Rooted in Løgstrup’s ethics of trust and human vulnerability (Løgstrup, 1997), Martinsen highlights the ethical asymmetry inherent in caring relations (Martinsen, 2012). Her engagement with Foucault’s analyses of institutional power sensitises attention to how organisational structures and coercive frameworks shape everyday nurse–patient interactions (1991; Foucault, 1980). These perspectives inform the interpretative phase of the analysis rather than functioning as predetermined analytical categories.

### Rational

Forensic psychiatry, characterised by its dual governance, creates ongoing tensions between rehabilitation aims, patient autonomy, and institutional control. While health promotion is increasingly emphasised within recovery-oriented frameworks, there remains limited empirical insight into how nurses understand and enact health promotion when their relational work is embedded in coercive routines, security requirements, and structural constraints.

A closer examination is therefore needed of how health-promoting practices are negotiated within the moral and institutional conditions of forensic psychiatric care. The aim of this study was to explore registered nurses’ experiences of promoting health and supporting everyday life in forensic psychiatric care.

Research question:

How do registered forensic nurses experience and navigate health promotion within the relational and structural conditions of forensic psychiatric care?

## Method

### Design

This exploratory qualitative study used reflexive thematic analysis (Braun & Clarke, 2006), drawing on care ethics and theories of institutional power to guide the interpretation.

### Research setting and procedure

The study was conducted at two forensic psychiatric hospitals in Sweden, with approximately 340 staff members and approximately 200 patients. The participating site represented a medium-secure context in which nurses balance strict security regulations with therapeutic and health-promoting interventions. Most patients in these settings were men aged between 25 and 45 years, most of whom had been convicted of violent offences. Approximately sixty per cent were diagnosed with schizophrenia or another psychotic disorder, often with comorbid substance use. An invitation to participate in the study, along with written information and a consent form, was distributed to the clinic’s heads and to each ward's head. Institutional approval for the study was granted by the clinic’s management.

### Participants

A purposive sample of fifteen registered nurses (*n*  =  15) working in forensic psychiatric care in Sweden participated. The sample size was guided by the concept of information power, which emphasised that the more relevant information participants possess regarding the study aim, the fewer participants are required (Malterud et al., 2016).

The participants represented diverse professional backgrounds and years of clinical practice, which helped capture a broad range of perspectives. The sample included nine women and six men aged 30–68 years. Participants’ professional experience ranged from five to over 45 years, with five to 30 years in forensic psychiatric care. All participants were employed at medium-secure forensic psychiatric clinics.

### Data collection

Individual face-to-face semi-structured interviews were conducted with registered nurses recruited through purposive sampling to ensure variation in professional experience and workplace context.

BB and CC collected interview data under the supervision of the first author (AA) between April and May 2025. All interviews were conducted in a quiet room at the participants’ workplaces, lasted 30–60 minutes, were audio recorded with consent, and were transcribed verbatim. The interview guide focused on nurses’ experiences supporting patients’ everyday lives, promoting health, and balancing care and safety requirements. The interview guide was used flexibly, and follow-up and clarifying questions were posed when appropriate to encourage participants to elaborate on their experiences.

### Data analysis

The interview transcripts were analysed using reflexive thematic analysis, following the six-phase approach described by Braun & Clarke, (2006;2021).

The first phase involved familiarisation with the data, during which the first author (AA) read and re-read all transcripts to gain an overall understanding of the material. In the second phase, coding was conducted inductively, based on participants’ own expressions and meanings emerging from the data rather than on predetermined categories. The first, second and third authors each independently coded one interview. These initial codings were then compared and discussed, and a shared coding approach was agreed upon. Guided by this approach, the first author subsequently coded two additional interviews, the second author coded six, and the third author coded the remaining six interviews. Throughout this process, the authors jointly reviewed and refined the codes through ongoing discussion, resulting in a final set of analytically developed codes.During the third phase, we searched for themes, examined codes for patterns of shared meaning, and clustered them into preliminary themes.

The fourth phase involved a critical review of these preliminary themes in relation to both the coded data extracts and the dataset as a whole. The coherence, boundaries and analytic focus of each theme were discussed and refined. During this phase, some themes were merged or reframed to enhance conceptual clarity. For example, the preliminary themes “*Supporting motivation”* and “*Encouraging participation”* were integrated into the overarching theme “*Dialogue as a foundation for participation”*. Similarly, the preliminary theme “*Health promotion in everyday care”* was reframed as “*Everyday practices as vehicles for recovery”* to better reflect its interpretative focus.

In the fifth phase, the thematic structure was further refined and consolidated into overarching themes. Attention was given to articulating the central organising idea of each theme and clarifying how the themes related to the study aim. Themes were defined and named to capture their core analytic meaning rather than to summarise content. The sixth phase, producing the paper, was integrated throughout the analytic process, with writing treated as an analytic activity through which the themes were further refined and contextualised.

In line with Braun and Clarke’s reflexive thematic analysis (2006; 2021), the analysis was understood as an iterative and interpretative engagement with the data rather than a purely inductive process. While the initial coding and development of themes were informed by participants’ accounts, theoretical perspectives were used reflexively to deepen interpretation and support sense-making at later stages of the analysis. Perspectives from care ethics and analyses of institutional power sensitised the analytic process by shaping how meanings, tensions and patterns in the data were interpreted, without functioning as predefined coding categories.

An ongoing reflexive dialogue among co-authors contributed to complementary academic perspectives that challenged interpretations and enhanced analytical depth, rather than achieving coding consensus. The final themes are presented in the Results section and illustrated with representative quotations.

To enhance transparency, Table I presents an illustrative example of the analytic process, showing how meaning units were condensed into initial codes, organised into subthemes, and synthesised into overarching themes. The excerpts included in the table are drawn from the interview transcripts but differ from those presented in the Results section to avoid unnecessary repetition.

**Table I.** Example of the analytical process from a meaning unit to a theme.

Meaning unit (data extract)	Initial code	Subtheme	Theme
*“You try to encourage the patient to take part in activities, but often they lack motivation. Then it becomes our task to find small things that can give some meaning in everyday life.”* (D7)	Motivation as a nursing task	Motivational dialogues support patients’ own motivation for change	Dialogue as a foundation for participation
*“Even something as simple as a daily walk outside changes the atmosphere. Patients talk more freely outdoors than they do on the ward.”* (D4)	Outdoors promotes openness	Physical activity and small steps	Everyday practices as vehicles for recovery
*“We talk about exercise and diet, but at the same time we give medication that causes weight gain. Patients see the contradiction and sometimes lose trust in what we say.”* (D11)	Medication undermines health advice	Paradox of medicines and health promotion	Navigating paradoxes within restrictive care

### Researcher reflexivity

The research team shared similar professional backgrounds: all were registered nurses; two had extensive clinical experience in psychiatric nursing (BB & CC), three in forensic nursing (DD, EE & FF), and one in health promotion and public health (AA). Academically, all authors held specialist psychiatric-mental health nursing half-master's and three a PhD (BB CC & DD), one of whom was a professor of nursing science (CC). This diversity provided complementary perspectives that enriched the study design, data interpretation, and discussion, while also necessitating continuous reflexivity to minimise the influence of pre-understandings. Reflexive discussions were held throughout the study to examine assumptions, challenge differing viewpoints, and ensure that the analysis was grounded in participants’ accounts rather than researchers’ expectations. At the same time, we acknowledge that the shared professional background of the research team also entails potential blind spots, including the risk of normalising institutional practices or underexploring tensions related to power, coercion, and control in forensic psychiatric care. To address this, reflexive discussions explicitly focused on identifying ambiguities, tensions, and discomforts in the data, as well as on considering alternative interpretations that challenged dominant professional narratives.

We also recognise the absence of lived experience perspectives within the research team. This constitutes a limitation of the study and is reflected in the discussion, where we consider how the lack of service-user perspectives may have shaped the analytical focus and the interpretation of the findings.

### Ethical considerations

The study was conducted in accordance with the Declaration of Helsinki (World Medical Association, 2013). Participation was voluntary; all participants received written and oral information about the study and provided written informed consent. Confidentiality was ensured by removing identifying details from transcripts, and participants were informed that they could withdraw at any time without consequences. Study approved by [redacted].

## Results

The result is reflected in three overarching themes that captured nurses’ experiences of promoting health and supporting everyday life in forensic psychiatric care: Dialogue as a foundation for participation, Everyday practices as vehicles for recovery, and Navigating paradoxes within restrictive care (please see Table II). Together, these themes illustrated the relational, practical, and structural dimensions of health promotion in a coercive care context.

**Table II.** Presents the themes and subthemes derived from the reflexive thematic analysis.

Themes	Subthemes
Dialogue as a foundation for participation	Building relationships despite coercion Motivational dialogue to support patients’ own motivation for change Small everyday choices as a sense of control and dignity
Everyday practice as a vehicle for recovery	Physical activity and small steps Nutrition and shared meals Everyday structure as invisible medicine
Navigating paradoxes within restrictive care	Psychoeducation for empowerment Paradox of medication and health promotion Balancing care and security obligations

### Dialogue as a foundation for participation

#### Building relationships despite coercion

Dialogue was described as a central but often fragile tool for creating trust and enabling patient participation. Nurses emphasised that conversations were not merely about transferring information but about establishing relationships that fostered safety and agency, while also being shaped by institutional routines and security requirements even within a coercive context.

*“If we cannot create a relationship where the patient feels safe and respected, then it is difficult to help them make any changes. We must meet them where they are, not where we want them to be.”* (D3)

Building relationships was therefore described as an important condition for health-promoting work, but not as something that could be taken for granted in everyday practice. The coercive nature of forensic psychiatric care often limited patients’ freedom, yet nurses sought to counterbalance this by cultivating trustful dialogues. By meeting patients “where they are, they aimed to acknowledge individual circumstances rather than impose predetermined expectations”.

#### Motivational dialogue to support patients’ own motivation for change

In addition to building relationships, nurses characterised motivational dialogue as necessary for supporting patients’ engagement in change, but also as work that required careful negotiation within coercive and institutional constraints. Rather than instructing patients, they emphasised active listening and encouraged them to reflect on their own reasons for change, while recognising that patients’ motivation could fluctuate and be influenced by detention conditions.

*“It’s about really listening actively and creating a dialogue where the patient feels heard. Many need to find their own reasons to change, not just be told why it is good.”* (D8)

Through such dialogue, nurses sought to support patients’ participation in health-promoting activities on their own terms, although participants also described limits to how far such dialogue could influence change when institutional demands took precedence.

#### Small everyday choices as a sense of control and dignity

Nurses also highlighted how offering patients small choices in everyday life could support participation within restrictive conditions, even though the scope for such choices was often narrow and tightly regulated. Even minor decisions were described as meaningful for patients’ sense of control.

*“Sometimes it can be as simple as letting them decide whether to go for a walk before or after lunch. These small things may not seem important, but they give a sense of control and dignity.”* (D6)

Providing opportunities for choice was described as a way of counteracting the loss of autonomy often experienced in forensic psychiatric care, while also reflecting the careful balancing of care, control, and institutional responsibility in everyday practice. Such opportunities were further seen as an important aspect of supporting participation in everyday life.

### Everyday practice as a vehicle for recovery

#### Physical activity and small steps

Health promotion was closely linked to supporting patients in developing healthier lifestyle habits, with physical activity often regarded as a practical starting point rather than the starting point. Nurses emphasised that even small, simple activities could be meaningful, for both physical health and building patients’ confidence, particularly within the constraints of everyday forensic psychiatric care.

*“It can be taking a walk around the house. It doesn’t have to be big. The important thing is to get the body moving and to feel capable of doing something.”* (D4)

This highlighted how nurses worked to break down health promotion into manageable, realistic goals for patients, adapted to patients’ current capacities and circumstances. The emphasis on “small steps” reflects an understanding that recovery is often fragile in this context and may be non-linear and requires individualised pacing. Physical activity was thus framed not only as a way of preventing illness but also as a tentative and situational therapeutic tool for strengthening self-esteem and everyday functioning.

#### Nutrition and shared meals

Another recurring aspect of health promotion concerned diet and nutrition. Many patients struggled with being overweight, having poor eating habits, or a lack of interest in food. Nurses described how involving patients in cooking or creating shared mealtime routines provided opportunities for both skill development and motivation, within the limits of institutional routines and security regulations.

*“When patients get to be part of cooking or at least choose something now and then—that often sparks interest. It’s not just something put in front of them.”* (D9)

Through these practices, nurses sought to transform meals from passive routines into participatory and social activities, although such opportunities were not always consistently available in everyday ward life. Cooking together or discussing food choices created opportunities for dialogue but also required careful coordination and negotiation within the institutional setting. At the same time, these practices supported a sense of community and normality within the ward. Nutrition was therefore described not only as a matter of physical health but also as a way to foster moments of meaning and social connection in an otherwise highly structured environment.

#### Everyday structure as “invisible medicine”

Nurses also emphasised the importance of establishing daily routines. Many patients entered care with disrupted sleep patterns, irregular eating habits, and little structure in their everyday lives. Creating predictable routines was considered fundamental to stability and recovery.

*“Many don’t know what day it is when they arrive. They don’t eat, don’t sleep at night, live in total disorder. Then structure is almost like medicine.”* (D8)

Here, everyday routines were described as a form of “invisible medicine” that supported both mental and physical well-being, while also being closely tied to institutional organisation and control. Regular mealtimes, sleep hygiene, and scheduled activities provided patients with a sense of rhythm and order, while also being shaped by ward routines and security requirements, thereby promoting self-esteem and participation. Nurses stressed that even small elements of routine could have significant effects on patients’ recovery, although these effects were described as gradual and sometimes fragile.

### Navigating paradoxes within restrictive care

#### Psychoeducation for empowerment

Nurses described psychoeducation as a central part of health-promoting work in forensic psychiatric care. Providing patients with information about illness, medication, and lifestyle was considered a means of increasing understanding and supporting participation in care, while also raising questions about the balance between empowerment and compliance.

Nurses emphasised the importance of adapting information to each patient’s level of comprehension and readiness, particularly given the complexity of patients’ conditions. Psychoeducation was described as an ongoing process rather than a one-time intervention, often integrated into everyday conversations and activities, and shaped by institutional expectations and treatment requirements.

*“We talk a lot with patients about why they take medication and what it does. They must understand, otherwise it’s hard for them to take responsibility for their health.”* (D11)

At the same time, some nurses reflected on the challenges of ensuring that psychoeducation supported patients’ own understanding and agency, rather than merely reinforcing treatment adherence within a coercive context.

#### Paradox of medication and health promotion

A central paradox described by nurses concerned the tension between promoting healthy lifestyles and administering medications associated with weight gain, fatigue, or metabolic side effects, within a context where medication adherence was often prioritised over lifestyle change. Nurses noted that patients often questioned these contradictions, particularly when encouraged to engage in physical activity or manage their weight.

*“We tell them to exercise and eat healthier, but at the same time, we give medications that make them gain weight or feel tired.”* (D5)

Nurses described that openly acknowledging these paradoxes was necessary to maintain trust and support patients’ motivation to engage in health-promoting activities, while also recognising that such openness did not always resolve patients’ frustration or the structural contradictions involved.

#### Balancing care and security obligations

Health promotion was also shaped by the need to balance caring responsibilities with security requirements, often within narrow margins for professional discretion. Nurses described how institutional rules, safety considerations, and legal constraints influenced what was possible in everyday practice.

*“We always have to think about security first, and sometimes that limits what we can do together with the patients.”* (D7)

Balancing care and security were described as an ongoing challenge, requiring nurses to continuously adapt health-promoting activities to individual patients and the restrictive context of forensic psychiatric care, while also acknowledging that some limitations could not be overcome through adaptation alone.

#### Summary of results

Taken together, the three overarching themes illustrated how registered nurses in forensic psychiatric care experience health promotion as a multidimensional and often fragile process shaped by relational, practical, and structural conditions. Dialogue emerged as a foundation for participation, encompassing relationship-building, motivational dialogue, and the use of small everyday choices to support patients’ sense of control and dignity within a coercive context, while also being constrained by institutional routines and security requirements. Everyday practices such as physical activity, shared meals, and structured routines were described as key vehicles for recovery, supporting stability, participation, and well-being in daily life, although such practices were often dependent on patients’ mental state, motivation, and the conditions of the institutional setting. At the same time, nurses described how health promotion involved navigating paradoxes within restrictive care, including tensions between medication and lifestyle advice, the ambivalent role of psychoeducation, and the ongoing need to balance caring responsibilities with security obligations and legal constraints.

**Figure 1.**
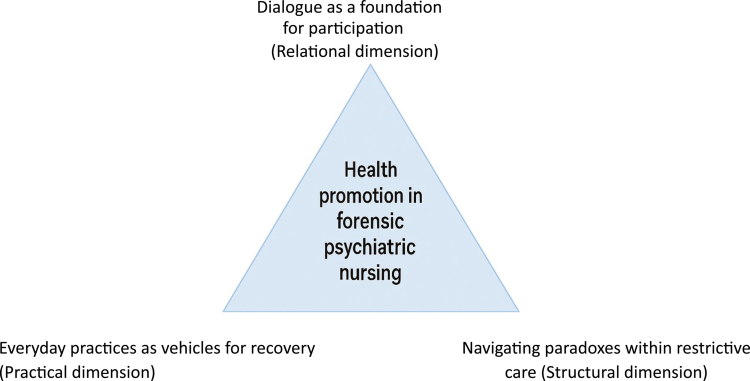
Conceptual model of health promotion in forensic psychiatric care, illustrating three interrelated dimensions of nursing work: relational (dialogue as a foundation for participation), practical (everyday practices as vehicles for recovery), and structural (navigating paradoxes within restrictive care).

Figure 1 provides an overview of the themes, illustrating how nurses’ experiences of health promotion in forensic psychiatric care are organised into three overarching themes reflecting the relational, practical, and structural dimensions of their work.

## Discussion

This study explored registered forensic nurses’ experiences of promoting health and supporting everyday life in forensic psychiatric care. Nurses described health promotion work as relational and practical work that is simultaneously shaped and constrained by structural and institutional conditions. By focusing on registered nurses’ perspectives, the study addresses a relative gap of attention in previous research, which has primarily examined treatment outcomes, organisational conditions, or patient experiences in forensic psychiatry (Merkt et al., 2021; Tully et al., 2024). The findings show how dialogue, everyday practices, and ethical, organisational paradoxes intersect to shape possibilities for health promotion under coercive conditions, rather than offering straightforward solutions.

### Dialogue as the core of relational nursing

Dialogue emerged as a foundational element of health-promoting nursing practice in forensic psychiatric care, yet one that was continually shaped and limited by institutional and coercive conditions. Nurses described relationship-building, motivational dialogue, and the provision of small everyday choices as key relational strategies for fostering trust and engagement despite coercive conditions. Rather than functioning merely as a means of information exchange, dialogue was experienced as a relational practice through which patients could feel respected, recognised, and involved in their own care while remaining embedded in asymmetrical power relations inherent to philosophy of care (Martinsen, 1993). While nurses described dialogue as supportive and trust-building, their role simultaneously involves enforcing institutional rules, thereby positioning them as both caregivers and agents of control (Hammarström et al., 2025).

These findings align with previous research emphasising the importance of therapeutic alliances for recovery in forensic settings (Magnusson et al., 2020). Recent Nordic studies similarly highlight how dialogue creates micro-opportunities for participation and humanisation within restrictive security structures (Sollied & Kvande, 2025). In the present study, the opportunity to make small choices, such as deciding when to take a walk or participating in everyday decisions, was described as particularly meaningful, yet also limited in scope and contingent on institutional approval, underscoring that dialogue is enacted not only verbally but also through everyday interactions. From Martinsen’s perspective, such everyday interactions may be understood as morally significant practices in which care is expressed through attentiveness and situated judgement in concrete care situations (Martinsen, 2006).

From a theoretical perspective, the findings of this study resonate with Martinsen’s philosophy of care, which emphasises trust, responsibility, and ethical attentiveness in asymmetrical caring relationships (Martinsen, 2012). Drawing on Løgstrup’s understanding of trust as a fundamental ethical condition (Løgstrup, 1997), the nurses’ relational work can be understood as a response to patients’ vulnerability and dependence, while simultaneously placing nurses in positions of heightened moral responsibility within coercive settings. Which aligns with Martinsen’s understanding of care as grounded in human interdependence and in a responsibility to respond to the other’s vulnerability (Martinsen, 2000). Dialogue thus becomes not only a relational strategy but also a fragile and contested condition for health-promoting work in contexts where patients’ opportunities for autonomy are structurally limited. Through this theoretical lens, everyday relational practices described by the nurses can be reinterpreted as situated negotiations between ethical responsiveness and institutional regulation. At the same time, relational engagement does not take place outside institutional systems of surveillance and control. Even well-intentioned dialogue remains circumscribed by legal mandates and security frameworks, raising questions about how far participation and autonomy can be realised within coercive care. While ethically meaningful, relational engagement does not fundamentally alter the structural asymmetry between nurse and patient; rather, participation is granted within boundaries defined by institutional logics of security and risk management rather than negotiated on equal terms. This tension also reflects Martinsen’s observation that rules and norms may guide practice without necessarily resolving what ought to be done in ethically complex care situations (Martinsen, 2006). This suggests that “small choices”, such as deciding when to take a walk, may function not only as expressions of relational care but can also be interpreted as instances of disciplinary power, where autonomy is permitted only within predefined institutional limits Foucault(1991, 1980). From this perspective and in line with previous research (Hammarström et al., 2019) dialogue emerges as both an ethical practice of care and a mechanism through which institutional order is maintained, highlighting the complex interplay between care, control, and responsibility in forensic psychiatric nursing.

### Everyday practices drive recovery in forensic care

The second theme highlights how health promotion was embedded in everyday practices, including physical activity, shared meals, and structured routines. Nurses described these practices as functioning as “invisible medicine”, providing stability, predictability, and a sense of mastery in everyday life, while simultaneously being embedded in institutional routines and expectations (Martinsen, 2005). In forensic psychiatric care, where autonomy is restricted and everyday life is highly regulated, such practices gain particular significance as concrete opportunities for recovery and participation, albeit within clearly defined boundaries.

These findings can be understood in relation to research on restrictiveness in forensic psychiatric care, which shows that everyday activities often constitute key sites where agency, autonomy, and control are negotiated within institutional constraints (Tomlin et al., 2018., Hellzén et al., 2023). From a recovery perspective, everyday practices such as walking, cooking, and maintaining routines may support connectedness, meaning, and empowerment, but also reflect normative expectations regarding appropriate behaviour and self-regulation. In forensic psychiatric care, such recovery processes must be understood within a safety-oriented context, as discussed in adaptations of the CHIME framework for forensic settings (Senneseth et al., 2021).

The nurses’ emphasis on small steps and manageable goals further illustrates their understanding of recovery as a fragile, non-linear process. Rather than focusing on behavioural change as a goal in itself, everyday practices were framed as ways to foster confidence, participation, and a sense of normality in daily life, even when broader constraints limited the scope for change. In this way, health promotion was closely intertwined with supporting patients’ everyday functioning and well-being under restrictive conditions (Hellzén et al., 2023). While such practices may foster stability and confidence, they may also reproduce normative expectations of compliance and self-regulation within a highly regulated environment. Everyday routines therefore operate both as supportive interventions and as mechanisms for maintaining institutional order (Martinsen, 2005). In this sense, everyday practices may also be interpreted as subtle forms of governance, where participation and self-management are encouraged within established frameworks of safety and order. Through this lens, recovery-oriented practices become embedded in institutional logics that simultaneously enable and regulate autonomy.

Participants rarely questioned their position of authority over patients, illustrating how everyday care practices remain embedded within institutional power relations. Rather than representing a straightforward movement towards empowerment, everyday care can thus be understood as situated within ongoing negotiations between support, risk management, and institutional control (Hammarström & Andreassen Devik, 2025). From this perspective, the institutional constraints described by participants may also reflect forms of disciplinary power that shape what becomes possible within caring encounters (1991; Foucault, 1980).

### Restrictive care demands navigating paradoxes

The third theme illuminates the paradoxes inherent in promoting health within forensic psychiatric care. Nurses described structural tensions between encouraging healthy lifestyles and administering medications associated with weight gain, fatigue, and metabolic side effects, within treatment regimes where pharmacological stability was often prioritised. From a Martinsens perspective, such situations highlight the importance of professional judgement in navigating ethically complex care practices where institutional rules cannot fully determine what ought to be done in the encounter with the patient (Martinsen, 2012). These findings are consistent with previous research highlighting ethical challenges and contradictory demands in psychiatric and forensic contexts (Hem et al., 2018; Jacob & Holmes, 2011). Open acknowledgement of these contradictions was described as essential for maintaining trust and supporting patients’ motivation, although such openness did not necessarily resolve patients’ experiences of inconsistency or frustration Which is in accordence with Martinsen’s understanding of care as requiring ethical attentiveness and the willingness to be touched by the other’s situation, where trust emerges through openness and responsiveness in the caring relationship (Martinsen, 2000).

The complexity of patients’ conditions, including psychotic disorders, substance use, neurodevelopmental conditions, and intellectual disabilities, further complicates health-promoting work. Recent Swedish studies have shown how that such comorbidities contribute to substantial health inequalities and challenges in forensic psychiatric populations (Noland et al., 2024; Sitter et al., 2025; Sivak et al., 2023). Within this context, nurses describe psychoeducation as a central strategy for supporting understanding and participation, while also raising questions about the balance between empowerment and treatment adherence, and recognising the limits imposed by cognitive impairments and institutional constraints. (Revelj et al., 2025).

The findings of this study suggest that these paradoxes place nurses in a position of continuous ethical negotiation much i line with Kari Martinsen (2005) reasoning. Health promotion, therefore, emerges as morally meaningful yet structurally constrained work, embedded within institutional arrangements that prioritise risk management and pharmacological stability. This structural positioning limits registered nurses’ discretionary space, shaping not only what can be done, but how health promotion can be framed and justified within a security-oriented logic. While nurses strive to promote participation and well-being, the structural logic of forensic care ultimately limits how far such efforts can extend.

These tensions must also be understood in light of the well-documented physical health inequalities and premature mortality among people with severe mental illness (Chan et al., 2023; Walker et al., 2015). Registered nurses are often acutely aware of long-term health risks related to weight gain, metabolic side effects of medication, smoking, and sedentary lifestyles (Lindskog et al., 2024). However, opportunities to address such risks may be limited by institutional routines, security regulations, restricted access to independent activity, and standardised food provision. Moreover, cognitive impairments and neurodevelopmental conditions may further complicate patients’ capacity to sustain lifestyle change (Hofvander et al., 2023; Wyler et al., 2024). Health promotion in this context, therefore, involves navigating not only ethical and organisational paradoxes, but also structural and cognitive constraints that may counteract preventive ambitions.

From a theoretical perspective, these tensions may also be understood as reflecting the dual character of forensic psychiatric care, where efforts to promote well-being are continuously negotiated within institutional mandates of control and risk management. Health-promoting work thus becomes situated within institutional power structures that simultaneously enable care while delimiting the scope of patient autonomy (Foucault, 1991).

### Dignity within structural and organisational constraints

Although dignity is a core nursing value, upholding it in practice is not always straightforward; structural and organisational conditions can make it difficult. This issue has received limited attention in psychiatric nursing research (Plunkett & Kelly, 2021), yet the present study shows that preserving dignity through everyday autonomy was a recurring concern for nurses working in coercive settings, often within narrow margins for action. Their efforts must be understood within healthcare systems characterised by limited staffing, high workloads, and institutional constraints. This also reflects Martinsen’s understanding of care as grounded in human vulnerability and interdependence, where the professional responsibility to respond to the other’s needs emerges within inherently asymmetrical caring relationships (Martinsen, 1993). In the present study, dignity did not emerge as an abstract principle but as something enacted through health-promoting practices, such as facilitating participation, enabling small choices, and structuring everyday life within coercive conditions.

Previous research in forensic psychiatric care has shown that maintaining patient dignity is a central yet challenging aspect of nursing practice, involving protection, respect, and recognition of the patient as a person despite restrictive and risk-oriented conditions (Gustafsson et al., 2013). Research on missed or unfinished nursing care further indicates that understaffing and workload intensity are associated with essential aspects of care being left undone (Gehri et al., 2024; Joseph et al., 2022). Against this backdrop, the health promotion strategies identified in this study, dialogue, trust-building, everyday practices and micro-opportunities for autonomy, can be understood as situated and often fragile enactments of dignity. While such practices may support personhood and participation, they cannot fully offset the structural constraints that shape care delivery in coercive institutional settings (Hellzén et al., 2023). In this context, dignity appears less as an inherent condition and more as an ongoing accomplishment, dependent on relational effort and organisational conditions. With Martinsen’s understanding of dignity as something that is realised within caring relationships, where attentiveness, responsiveness, and moral responsibility shape how the vulnerable other is recognised in concrete care practices (Martinsen, 2006).

From a theoretical perspective, these tensions may also be understood as reflecting the dual character of forensic psychiatric care, where efforts to promote well-being are continuously negotiated within institutional mandates of control and risk management. Health-promoting work thus becomes situated within institutional power structures that simultaneously enable care while delimiting the scope of patient autonomy.

## Overall contribution and implications

Taken together, the findings conceptualise health promotion in forensic psychiatric care as relational, practical, and structurally constrained work. By foregrounding nurses’ experiences, this study extends existing understandings of recovery-oriented practice by demonstrating how health promotion is enacted within institutional limits rather than outside them. These findings do not suggest that health promotion overcomes structural inequities; rather, they illuminate how nurses attempt to work within these constraints. The findings also illustrate how nurses engage in moral and relational labour while navigating these structural conditions. Health promotion in forensic psychiatric settings cannot be reduced to lifestyle interventions or behavioural change alone. Instead, it emerges as a situated moral practice, shaped by dialogue, everyday routines, and the ongoing navigation of structural tensions between care, control, and security. Strengthening organisational conditions, interprofessional collaboration, and recognition of health promotion as core nursing work may enhance the sustainability of recovery-oriented practices under coercive conditions. This study contributes to a more nuanced understanding of health promotion in forensic psychiatric care as relationally enacted, institutionally negotiated, and structurally constrained nursing practice.

### Implications for practice

- The findings highlight the need to recognise health promotion as a core nursing responsibility in forensic psychiatric care, while ensuring that such work is supported at an organisational level.
- The findings suggest the importance of strengthening interprofessional collaboration to balance health promotion with security requirements.
- Acknowledge and address cognitive difficulties as a central barrier to participation.
- Consider the need for staff training in adapting health-promoting strategies for patients with neurodevelopmental conditions and functional impairments.
- Explore ways of increasing organisational flexibility to support everyday routines and lifestyle practices as vehicles for recovery within existing safety and legal frameworks.

### Future research

- Further research could explore patients’ perspectives on health promotion to complement nurses’ viewpoints.
- Future studies may examine how cognitive difficulties affect engagement in health-promoting activities.
- There is a need to evaluate tailored interventions addressing both relational and structural barriers.
- Comparative research across security levels and national contexts could help identify organisational conditions that best support recovery-oriented care.

### Methodological considerations

We employed a semantic-analytic approach in reflexive thematic analysis (RTA), focusing on the explicit content of participants’ accounts rather than on latent meanings. As a result, prioritising explicit accounts may have limited exploration of latent meanings, underlying assumptions, or power dynamics in nurses’ narratives. This approach was considered appropriate given the study’s aim of exploring registered nurses’ experiences of promoting health and supporting everyday life in forensic psychiatric care, while acknowledging that alternative analytic approaches might have generated different insights.

The research team’s professional experience can be understood as both a resource and a limitation, as it contributed contextual understanding and sensitivity to the empirical material. At the same time, the research team’s close involvement across all stages of the study may have influenced the analytic process through shared pre-understandings, despite ongoing reflexive discussions aimed at challenging assumptions and alternative interpretations. The analysis was conducted over time, in line with the iterative and reflexive principles described by Braun & Clarke, (2006, 2021), allowing repeated engagement with the data and the refinement of themes. The data were collected at two forensic psychiatric clinics, representing approximately one-third of all Swedish forensic psychiatric clinics, a strength in terms of contextual breadth. At the same time, although this may enhance transferability within the national context, the findings may be less applicable to forensic psychiatric settings organised under different legal or organisational frameworks.

## Supplementary Material

Supplementary Material Semi structured interview guide to reviewer.docx

## Data Availability

Due to the sensitive nature of the study and the risk of compromising participant confidentiality, the interview data are not publicly available. The participants did not provide consent for their data to be shared beyond the research team.

## References

[cit0001] Braun, V., & Clarke, V. (2006). Using thematic analysis in psychology. *Qualitative Research in Psychology*, *3*(2), 77–101. 10.1191/1478088706qp063oa

[cit0002] Braun, V., & Clarke, V. (2021). One size fits all? What counts as quality practice in (reflexive) thematic analysis? *Qualitative Research in Psychology*, *18*(3), 328–352. 10.1080/14780887.2020.1769238

[cit0003] Chan, J. K. N., Correll, C. U., Wong, C. S. M., Chu, R. S. T., Fung, V. S. C., Wong, G. H. S., Lei, J. H. C., & Chang, W. C. (2023). Life expectancy and years of potential life lost in people with mental disorders: A systematic review and meta-analysis. *EClinicalMedicine*, *65*, 102294. 10.1016/j.eclinm.2023.102294 37965432 PMC10641487

[cit0004] Crump, C., Winkleby, M. A., Sundquist, K., & Sundquist, J. (2013). Comorbidities and mortality in persons with schizophrenia: A Swedish national cohort study. *Am J Psychiatry*, *170*(3), 324–333. 10.1176/appi.ajp.2012.12050599 23318474

[cit0005] Foucault, M. (1980). In C. Gordon (Ed.), *Power/knowledge: Selected interviews and other writings 1972–1977*. Pantheon Books.

[cit0006] Foucault, M. (1991). Governmentality. In I. G. Burchell, C. Gordon, & P. Miller (Eds.), *The Foucault effect: Studies in governmentality* (pp. 87–104). University of Chicago Press.

[cit0007] Gehri, B., Ausserhofer, D., Zúñiga, F., Bachnick, S., Schwendimann, R., & Simon, M. (2024). Nursing care left undone in psychiatric hospitals and its association with nurse staffing: A cross-sectional multi-centre study in Switzerland. *J Psychiatr Ment Health Nurs*, *31*(2), 215–227. 10.1111/jpm.12978 37697908

[cit0008] Gustafsson, L. K., Wigerblad, A., & Lindwall, L. (2013). Respecting dignity in forensic care: The challenge faced by nurses of maintaining patient dignity in clinical care situations. *J Psychiatr Ment Health Nurs*, *20*(1), 1–8. 10.1111/j.1365-2850.2012.01895.x 22417206

[cit0009] Hammarström, L., & Andreassen Devik, S. (2025). Navigating the ethical "space in-between" nurses' lived experiences in forensic inpatient care interpreted through Løgstrup's ethical philosophy. *Int J Qual Stud Health Well-being*, *20*(1) 2514520. 10.1080/17482631.2025.2514520 40471827 PMC12265931

[cit0010] Hammarström, L., & Andreassen Devik, S. (2025). Navigating the ethical “space in-between” nurses’ lived experiences in forensic inpatient care interpreted through Løgstrup’s ethical philosophy. *International Journal of Qualitative Studies on Health and Well-being*, 20(1), 2514520. 10.1080/17482631.2025.2514520 40471827 PMC12265931

[cit0011] Hammarström, L., Häggström, M., Devik, S. A., & Hellzen, O. (2019). Controlling emotions: nurses' lived experiences caring for patients in forensic psychiatry. *Int J Qual Stud Health Well-being*, *14*(1) 1682911. 10.1080/17482631.2019.1682911 31645227 PMC6818121

[cit0012] Hellzén, O., Hammarström, L., Ekman, O., & Devik, S. A. (2023). A meta-ethnographic review of forensic psychiatry inpatient care. Nursing staff experiences of the nurse-patient encounter. *Issues Ment Health Nurs*, *44*(12), 1226–1236. 10.1080/01612840.2023.2259997 37801705

[cit0013] Hem, M. H., Gjerberg, E., Husum, T. L., & Pedersen, R. (2018). Ethical challenges when using coercion in mental healthcare: A systematic literature review. *Nurs Ethics*, *25*(1), 92–110. 10.1177/0969733016629770 26931767

[cit0014] Hofvander, B., Nilsson, T., Ståhlberg, O., Claesdotter, E., Moberg, P., Ahlbäck, K., & Hildebrand Karlén, M. (2023). Autism spectrum disorders in forensic psychiatric investigations-patterns of comorbidity and criminality. *Front Psychiatry*, *14*, 1168572. 10.3389/fpsyt.2023.1168572 37621970 PMC10444990

[cit0015] Jacob, J. D., & Holmes, D. (2011). Working under threat: Fear and nurse-patient interactions in a forensic psychiatric setting. *J Forensic Nurs*, *7*(2), 68–77. 10.1111/j.1939-3938.2011.01101.x 21635678

[cit0016] Joseph, B., Plummer, V., & Cross, W. (2022). Mental health nurses perceptions of missed nursing care in acute inpatient units: A multi-method approach. *Int J Ment Health Nurs*, *31*(3), 697–707. 10.1111/inm.12990 35294094 PMC9314997

[cit0017] Lindskog, A., Lindroth, M., Holmgren, K., & Gunnarsson, A. B. (2024). Balancing on a slack line—Staffs’ experiences of talking about sexuality and sexual health with patients cared for in forensic psychiatry in Sweden. *Frontiers in Psychiatry*, *15*, 1450377. 10.3389/fpsyt.2024.1450377 39290296 PMC11406074

[cit0018] Løgstrup, K. E. (1997). *The ethical demand*. University of Notre Dame Press.

[cit0019] Lumén, K., Louheranta, O., & Kuosmanen, L. (2024). Forensic psychiatric Patients' experiences of personal recovery: A wilsonian concept analysis. *J Forensic Nurs*, *20*(2), 103–112. 10.1097/jfn.0000000000000477 38315513 PMC11882176

[cit0020] Lutz, M., Zani, D., Fritz, M., Dudeck, M., & Franke, I. (2022). A review and comparative analysis of the risk-needs-responsivity, good lives, and recovery models in forensic psychiatric treatment. *Front Psychiatry*, *13*, 988905. 10.3389/fpsyt.2022.988905 36386990 PMC9659584

[cit0021] Magnusson, E., Axelsson, A. K., & Lindroth, M. (2020). We try' - how nurses work with patient participation in forensic psychiatric care. *Scand J Caring Sci*, *34*(3), 690–697. 10.1111/scs.12773 31749183

[cit0022] Malterud, K., Siersma, V. D., & Guassora, A. D. (2016). Sample size in qualitative interview studies: Guided by information power. *Qual Health Res*, *26*(13), 1753–1760. 10.1177/1049732315617444 26613970

[cit0023] Martinsen, K. (1993). *Fra Marx til Løgstrup*. Tano.

[cit0024] Martinsen, K. (2000). Øyet og kallet, Bergen: Fagbokforlaget.

[cit0025] Martinsen, K. (2005). *Samtalen, skjønnet og evidensen [In Norwegian]*. Oslo: Akribre.

[cit0026] Martinsen, K. (2006). Care and vulnerability, Oslo: Akribe Cappelen Damm AS. https://books.google.se/books?id=pla0GAAACAAJ.

[cit0027] Martinsen, K. (2012). Løgstrup and nursing. *Nursing Philosophy*, *13*(3), 215–224. 10.1111/j.1466-769X.2011.00514.x

[cit0028] Merkt, H., Haesen, S., Eytan, A., Habermeyer, E., Aebi, M. F., Elger, B., & Wangmo, T. (2021). Forensic mental health professionals’ perceptions of their dual loyalty conflict: Findings from a qualitative study. *BMC Medical Ethics*, *22*(1), 123. 10.1186/s12910-021-00688-2 34530830 PMC8444425

[cit0029] Noland, E., Virtanen, S., Klötz Logan, F., Chang, Z., & Strandh, M. (2024). Post-discharge pharmacological treatment discontinuation of forensic psychiatric patients in Sweden. Frontiers in Psychiatry, 15, 1342722. 10.3389/fpsyt.2024.1342722 38404465 PMC10884161

[cit0030] Patel, V., Saxena, S., Lund, C., Thornicroft, G., Baingana, F., Bolton, P., Chisholm, D., Collins, P. Y., Cooper, J. L., Eaton, J., Herrman, H., Herzallah, M. M., Huang, Y., Jordans, M. J. D., Kleinman, A., Medina-Mora, M. E., Morgan, E., Niaz, U., Omigbodun, O., … Sunkel, C. (2018). The lancet commission on global mental health and sustainable development. *The Lancet*, *392*(10157), 1553–1598. 10.1016/S0140-6736(18)31612-X 30314863

[cit0031] Plunkett, R., & Kelly, B. D. (2021). Dignity: The elephant in the room in psychiatric inpatient care? A systematic review and thematic synthesis. *Int J Law Psychiatry*, *75*. 101672. 10.1016/j.ijlp.2021.101672 33513475

[cit0032] Revelj, J., Hörberg, U., Wallinius, M., & Rask, M. (2025). Caregivers' experiences of providing care to female patients in Swedish forensic psychiatric care. *Frontiers in Psychiatry*, *16*. 1646726. 10.3389/fpsyt.2025.1646726 41268372 PMC12626913

[cit0033] Salzmann-Erikson, M., Rydlo, C., & Wiklund Gustin, L. (2016). Getting to know the person behind the illness - the significance of interacting with patients hospitalised in forensic psychiatric settings. *J Clin Nurs*, *25*(9-10), 1426–1434. 10.1111/jocn.13252 26997335

[cit0034] Sariaslan, A., Sharpe, M., Larsson, H., Wolf, A., Lichtenstein, P., & Fazel, S. (2022). Psychiatric comorbidity and risk of premature mortality and suicide among those with chronic respiratory diseases, cardiovascular diseases, and diabetes in Sweden: A nationwide matched cohort study of over 1 million patients and their unaffected siblings. *PLoS Med*, *19*(1), e1003864. 10.1371/journal.pmed.1003864 35085232 PMC8794193

[cit0035] Senneseth, M., Pollak, C., Urheim, R., Logan, C., & Palmstierna, T. (2021). Personal recovery and its challenges in forensic mental health: Systematic review and thematic synthesis of the qualitative literature. *BJPsych Open*, *8*(1). e17. 10.1192/bjo.2021.1068 34915963 PMC8715254

[cit0036] SFS 1991:1129. Lag (1991:1129) om rättspsykiatrisk vård. Stockholm: Socialdepartementet. Available from the Swedish Code of Statutes.

[cit0037] Sivak, L., Forsman, J., & Masterman, T. (2023). Duration of forensic psychiatric care and subsequent criminal recidivism in individuals sentenced in Sweden between 2009 and 2019 [Original Research]. *Frontiers in Psychiatry*, *14*, 2023. 10.3389/fpsyt.2023.1129993 PMC10053040 37009123

[cit0038] Sitter, T. M., Virtanen, S., Edberg, H., Andiné, P., Fernqvist, A., Noland, Ebba, Hirvikoski, T., Nilsson, T., & Chang, Z. (2025). Pharmacological treatment and psychiatric polypharmacy in forensic psychiatric care in Sweden. *European Psychiatry*, 68(1), e78. 10.1192/j.eurpsy.2025.10031 40495520 PMC12188328

[cit0039] Sollied, S. A., & Kvande, M. E. (2025). Humanization of care in forensic mental health wards: A qualitative study. *International Journal of Qualitative Studies on Health and Well-being*, *20*(1), 2448153. 10.1080/17482631.2024.2448153 41489220 PMC11703350

[cit0040] Thomson, L., & Rees, C. (2023). Long-term outcomes of the recovery approach in a high-security mental health setting: A 20-year follow-up study. *Frontiers in Psychiatry*, *14*, 1111377. 10.3389/fpsyt.2023.1111377 37252143 PMC10213922

[cit0041] Tomlin, J., Bartlett, P., & Völlm, B. (2018). Experiences of restrictiveness in forensic psychiatric care: Systematic review and concept analysis. *Int J Law Psychiatry*, *57*, 31–41. 10.1016/j.ijlp.2017.12.006 29548502

[cit0042] Tully, J., Hafferty, J., Whiting, D., Dean, K., & Fazel, S. (2024). Forensic mental health: Envisioning a more empirical future. *The Lancet Psychiatry*, *11*(11), 934–942. 10.1016/s2215-0366(24)00164-0 38945145

[cit0043] Walker, E. R., McGee, R. E., & Druss, B. G. (2015). Mortality in mental disorders and global disease burden implications: A systematic review and meta-analysis. *JAMA Psychiatry*, *72*(4), 334–341. 10.1001/jamapsychiatry.2014.2502 25671328 PMC4461039

[cit0044] World Health Organization Regional Office for Europe. (2025). *Mental health and well-being in prisons and other detention centres*. W. H. Organization. https://www.who.int/europe/publications/i/item/WHO-EURO-2025-10968-50740-76853

[cit0045] Wyler, H., van Wijnkoop, M., Smith, A., Retz, W., Liebrenz, M., & Buadze, A. (2024). Lost diagnoses? A multi-year trajectory of patients with childhood ADHD in the criminal justice system in Switzerland [Original Research]. *Frontiers in Psychiatry*, *15*, 2024. 10.3389/fpsyt.2024.1403618 PMC11187301 38903643

