# Supplementary material for: Health promotion as relational, practical, and structurally constrained work in forensic psychiatric care: a qualitative study on registered nurses’ experiences
Source: Int J Qual Stud Health Well-being. 2026 Apr 10;21(1):2657694. doi: 10.1080/17482631.2026.2657694 (PMC13072670; doi:10.1080/17482631.2026.2657694)
Supplement: Supplementary Material Semi structured interview guide to reviewer.docx [file ZQHW_A_2657694_SM3214.docx]

**Supplementary Material 1. Semi-structured interview guide**

The interviews were guided by a semi-structured interview guide focusing on registered nurses’ experiences of health promotion and everyday care in forensic psychiatric settings. The questions were used flexibly, allowing for follow-up questions and probes depending on participants’ responses. This is a translated version of the original Swedish interview guide.

**1. Background information**

- What is your professional education?
- How old are you?
- How do you describe your gender?
- How long have you worked in psychiatric and/or forensic psychiatric care?

**2. Work context**

- Can you describe your role as a registered nurse in forensic psychiatric care?
- How do your work tasks relate to health-promoting activities for patients under compulsory care?

**3. Experiences of health-promoting work**

- How do you experience working to support patients in improving their physical and mental health?
- What kinds of health-promoting activities or interventions do you typically engage in with patients?

**4. Challenges and opportunities**

- What do you experience as the main challenges in working with health promotion in compulsory forensic psychiatric care?
- Are there factors that facilitate or support your health-promoting work?

**5. Patient relationships and encounters**

- How do patients typically respond to health-promoting activities?
- In what ways does the context of compulsory care influence how you work with health promotion?

**6. Participation and collaboration**

- How do you involve patients in decisions related to their health and everyday life?
- How do you collaborate with colleagues and other professional groups to promote patients’ health?

**7. Reflection and development**

- Is there anything you would like to change or improve in the way health-promoting work is carried out?
- How do you view the future of health-promoting work in forensic psychiatric care?

**8. Closing questions**

- Is there anything you would like to add that we have not discussed?
- What do you consider to be the most important insight from your work with health promotion in forensic psychiatric care?
